# BoALG10, an α-1,2 glycosyltransferase, plays an essential role in maintaining leaf margin shape in ornamental kale

**DOI:** 10.1093/hr/uhac137

**Published:** 2022-06-15

**Authors:** Xin Feng, Xinru Yang, Meiqin Zhong, Xin Li, Pengfang Zhu

**Affiliations:** College of Forestry, Shenyang Agricultural University, Shenyang 110866, China; Key Laboratory of Forest Tree Genetics, Breeding and Cultivation of Liaoning Province, Shenyang 110866, China; College of Forestry, Shenyang Agricultural University, Shenyang 110866, China; College of Forestry, Shenyang Agricultural University, Shenyang 110866, China; College of Forestry, Shenyang Agricultural University, Shenyang 110866, China; College of Forestry, Shenyang Agricultural University, Shenyang 110866, China; Key Laboratory of Forest Tree Genetics, Breeding and Cultivation of Liaoning Province, Shenyang 110866, China

## Abstract

The morphological diversity of leaf margin shapes is an identifying characteristic of many plant species. In our previous work, BoALG10 (α-1,2 glycosyltransferase) was predicted to be a key regulator of leaf margin shape in ornamental kale (*Brassica oleracea* var. *acephala*). An alanine and a leucine residue in the conserved domain of the smooth-margined S0835 were replaced by an aspartate and a phenylalanine, respectively, in the corresponding positions of the feathered-margined F0819. However, the expression pattern and function of this gene remain unclear. Here, we examined the expression patterns of *BoALG10* using quantitative real-time PCR, and found that statistically significant differences in expression existed between F0819 and S0835 in nine developmental stages. The BoALG10 protein localized to the endoplasmic reticulum. The function of *BoALG10* was then examined using complementary mutant assays. The overexpression strains phenocopied the smooth leaf margin after introduction of *BoALG10*^*S0835*^ into the feathered-margined inbred line F0819. Simultaneously, irregular dissections appeared in the leaf margins of knockout mutants KO-1 and KO-2, which were generated by CRISPR/Cas9 technology from the smooth-margined inbred line S0835. Microscopic observation showed that the leaf margin cells of the smooth-margined plants S0835 and OE-3 were arranged regularly, while the cells of the feathered-margined plants F0819 and KO-1 were of inconsistent size and distributed in an irregular manner, particularly around the indentations of the leaf. This elucidation of *BoALG10* function provides a novel insight into the morphological regulation of leaf margin shape.

## Introduction

Leaves are important plant organs, providing a platform for gas exchange, water transport, and photosynthesis [1, 2]. There is huge variation in leaf size and shape, with several types of leaf margin, including lobed, serrated, and smooth edges [1]; this diversity is a key characteristic in the morphological identification of various species [3]. Serrated or deeply lobed leaf shapes can increase heat dissipation and enhance disease defense of white oak by increasing the distance between leaves [4]. The rounder leaves in tomato (*Solanum lycopersicum*) were recently shown to significantly improve the flavor and quality of the fruits [5].

Leaves originate from the shoot apical meristem [6], and their development, including margin formation, is regulated by many genes [7–12]. Members of the *YUCCA* gene family regulate leaf growth and margin development in response to auxin [13], while the *NAC* (*NAM*, *ATAF1/2*, and *CUC2*) family genes play an important role in controlling the formation of the shoot apical meristem in the embryo, and thus the establishment of leaf margins [14]. The role of *CUP-SHAPED COTYLEDON2* (*CUC2*) is essential for the early stages of leaf margin serration in *Arabidopsis thaliana* [9], and *CUC3* together with miR164A [15] maintains the serrated shape in later development [16, 17]. The transcription factors *NGATHA* and *CINCINNATA-class-TCP* (*CIN-TCP*) function redundantly to inhibit leaf margin development, leading to the production of crinkly and serrated leaf margins by restricting the activity of the shoot apical meristem [6]. Other TCP transcription factors, *TCP3*, *TCP4*, *NGA1*, and *NGA4*, regulated by miR319 were found to be involved in the development of the central and marginal regions of *Arabidopsis* leaves [7, 18]. *LATE MERISTEM IDENTITY 1* (*LMI1*) plays an important role in the formation of a serration margin in a simple leaf [19], while the Cys2His2 zinc finger transcription factor *PALM1* was the key positive regulator of leaflet formation in compound *Medicago truncatula* leaves [20]. Decreasing the expression of *REDUCED COMPLEXITY* (*RCO*) was shown to inhibit the growth of locally occurring lobules along the leaf margin, changing the leaf shape and producing deep-lobed leaves [21, 22] as well as negatively regulating multiple cytokinin-related genes to repress growth at the leaf base during leaf development [23].

Glycosyltransferases perform N-glycosylation, a post-translational protein modification involved in various biological processes [24–27]. N-glycosylation plays a positive role in the development of the cell wall [24], leaves [25], and roots [26]. α*-*1,2-Glucosyltransferase (ALG10) is a key enzyme that participates in the final step of liposaccharide formation, catalyzing the transfer of the last glucosyl molecule to a liposaccharide precursor to generate oligosaccharide chains in the N-glycan biosynthesis pathway [28]. From there, the oligosaccharide chains are catalyzed by the oligosaccharyltransferase complexes STT/OST (STAUROSPORIN AND TEMPERATURE SENSITIVE/oligosaccharyltransferase), which can recognize the nascent peptide chain glycosylation sites and transfer the oligosaccharide chains onto asparagine residues [25, 29, 30]. *ALG10* is known to play an important role in the normal development of plant leaves because its T-DNA insertion mutant produced dwarf leaves [25]; however, the mechanisms by which this occurred, and its effect on the maintenance of the leaf margin, are yet to be elucidated.

We previously predicted and cloned a putative leaf margin shape regulator, *BoALG10*, using forward genetics in the form of chromosome mapping in ornamental kale (*Brassica oleracea* var. *acephala*). There were two non-synonymous single-nucleotide polymorphisms (SNPs) in the conserved domain, which resulted in coding variations of an aspartate and a phenylalanine residue in a feathered leaf margin inbred line, F0819, but an alanine and a leucine residue in a smooth leaf margin inbred line, S0835 [31]. However, its expression and function remains unclear. In the present study, we detected the relative expression levels of *BoALG10* between F0819 and S0835 at different developmental stages to verify its temporal and spatial characteristics. We then constructed subcellular localization vectors to determine the location of the BoALG10 protein. In addition, to verify the function of *BoALG10* we built an overexpression vector harboring the S0835 allele *BoALG10^S0835^*, which was transformed into F0819. Moreover, a CRISPR/Cas9 knockout vector was transformed into S0835. Finally, we captured the microscopic structure of the epidermal cells at the leaf margin among the two inbred lines and their corresponding transgenic and genome-edited lines. The elucidation of the function of *BoALG10* in this work will provide new insights into leaf margin morphogenesis.

## Results

### The expression patterns of *BoALG10* showed significant differences between F0819 and S0835

**Figure 1:**
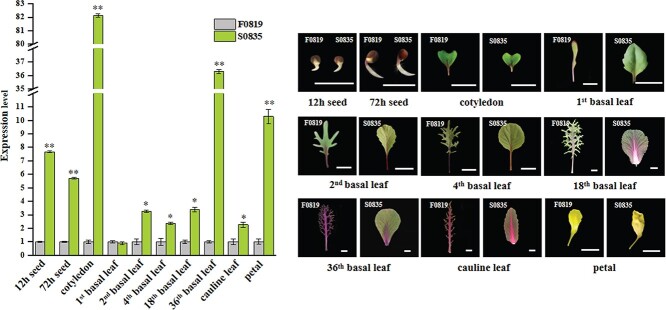
Relative expression of *BoALG10* between F0819 and S0835 in different developmental stages. **a** Relative expression level of *BoALG10* in 10 tissues of F0819 and S0835, determined using qRT–PCR. The 10 stages were: seeds after 12 and 72 hours of germination; cotyledons; 1st, 2nd, 4th, 18th, and 36th basal leaves; cauline leaves; and petals. *BoActin* served as a loading control, and F0819 was used as the reference sample. Relative expression level was calculated using the 2^–△△Ct^ method. Error bars represent the standard errors of three biological replicates. Asterisks represent a value significantly different from the reference, as determined using a *t*-test (^*^P < .05, ^**^P < .01). **b** Leaf phenotypes of F0819 and S0835 at the 10 developmental stages. Scale bars: 1 cm.

In a previous study, we fine-mapped and predicted a leaf margin candidate gene, *BoALG10*, using the inbred lines F0819 and S0835, which produce a feathered and a smooth leaf margin, respectively [31]. We isolated and sequenced the alleles of *BoALG10* in F0819 and S0835. There were two non-synonymous SNPs leading to two amino acid variations in the conserved domain in which an alanine and a leucine residue in the smooth leaf margin inbred line S0835 were replaced by an aspartate and a phenylalanine, respectively, in the corresponding positions of the feathered leaf margin inbred line F0819 [31]. Here, we examined the expression pattern of *BoALG10* between F0819 and S0835 in tissues from 10 developmental stages: seeds after 12 and 72 hours of germination; the cotyledons; basal leaves at five stages; cauline leaves; and petals (Fig. 1). *BoActin* was used as the internal control gene, and the expression level in F0819 was considered the reference value at each stage.

**Figure 2:**
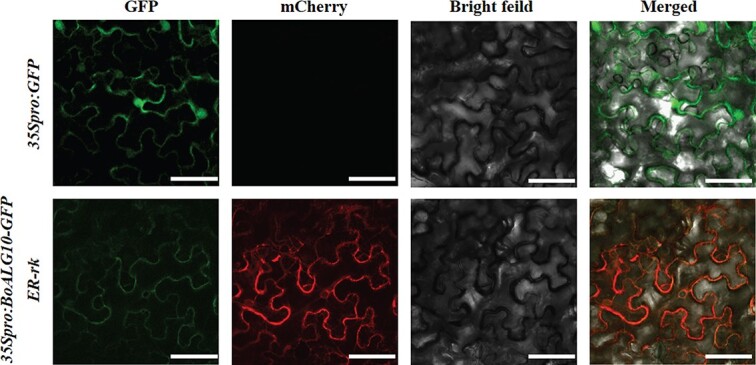
Subcellular localization of the BoALG10 protein. *35Spro:BoALG10*-*GFP* and *ER-rk* constructs were equally mixed and then infiltrated into 4-week-old *N. benthamiana* leaves, with the pCAMBIA1302 vector used as the blank control. Scale bars: 75 μm.

*BoALG10* was expressed at each developmental stage analyzed in both genotypes; however, significant differences in the relative level of *BoALG10* expression were apparent throughout development. The relative expression level of *BoALG10* was 7.67-fold higher in S0835 than in F0819 at the 12-hour seed stage, dropping to a 5.70-fold difference at the 72-hour seed stage. A huge difference in relative expression occurred in the cotyledon stage, when an 82.14-fold higher expression level in S0835 than F0819 was detected. When the first true leaf appeared, there was no significant difference between the *BoALG10* expression levels of S0835 and F0819. In the early rosette, the relative expression level was 3.27-, 2.36-, and 3.39-fold higher in the 2nd, 4th, and 18th basal leaves, respectively, of S0835 than of F0819. The second huge difference in relative expression occurred in the 36th basal leaf in the rosette, with a relative *BoALG10* expression level 36.65-fold higher in S0835 than in F0819. The relative expression decreased sharply to a 2.32-fold greater level in S0835 than in F0819 in the cauline leaves, while the petals of S0835 expressed *BoALG10* to a 10.31-fold higher level than those of F0819. All differences in expression were statistically significant except for those of the first basal leaf. We therefore inferred that *BoALG10* is expressed in all tissues throughout development.

### BoALG10 is localized to the endoplasmic reticulum

The subcellular localization of BoALG10 was analyzed by transiently expressing *35Spro:BoALG10*-*GFP* in *Nicotiana benthamiana* leaves. As shown in Fig. 2, the fluorescence signal was detected in the endoplasmic reticulum (ER), indicating presence of BoALG10 in the ER.

### Phylogenetic tree of putative BoALG10 homologs

**Figure 3:**
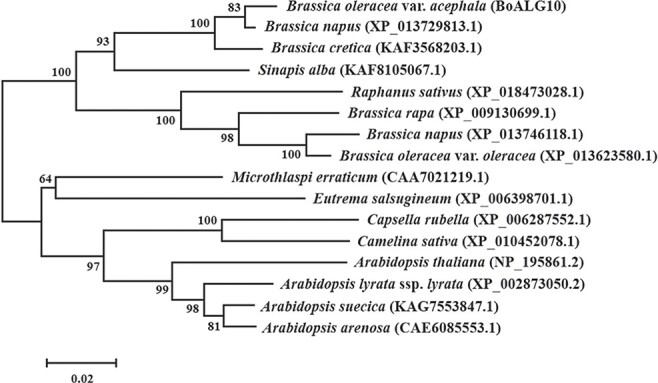
Phylogenetic tree of ornamental kale BoALG10 protein and its 15 homologs in Cruciferae species. The phylogram was constructed using the neighbor-joining method, using sequences obtained from NCBI. One thousand bootstrap replicates were performed, and the percentage of replicate trees in which the associated taxa clustered together is shown next to each branch. The evolutionary distances were computed using the Poisson correction method and are given in units of amino acid substitutions per site. All ambiguous positions were removed for each sequence pair.

To better understand the structure and evolution of BoALG10, we constructed a phylogenetic tree using the amino sequences of BoALG10 and its putative 15 homologs in Cruciferae (Fig. 3). The ALG10 protein was conserved with similarity of 93.27% in *Brassica* and 89.99% in Cruciferae (Fig. 3, Supplementary Data Figs S1 and S2). In *Arabidopsis*, *AtALG10* has been well characterized, and is known to be required for the biosynthesis of lipid-linked oligosaccharides and subsequently for normal leaf development [25].

### Overexpressing *BoALG10^S0835^* produces an S0835-like smooth leaf margin in F0819

**Figure 4:**
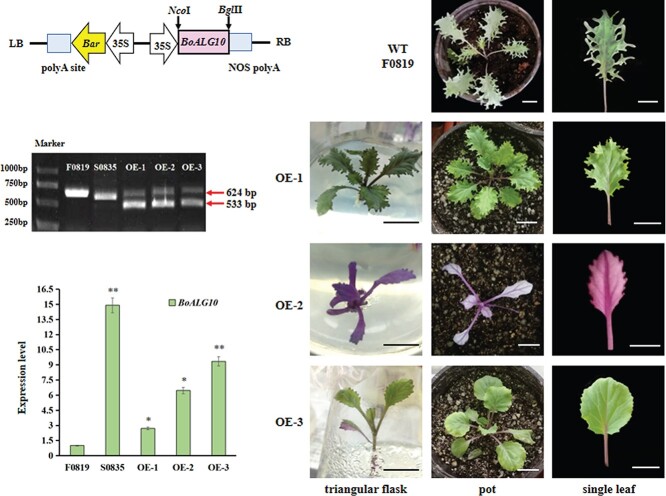
Generation and characterization of *BoALG10^S0835^*-overexpressing plants. **a** Schematic of the *BoALG10* overexpression cassette. **b** Phenotype of the WT F0819. Scale bars: 2 cm. **c** Phenotype of the transgenic *BoALG10^S0835^*-overexpressing lines. Scale bars: 2 cm. **d** PCR amplification of the T-DNA insert in the two parents and the transgenic lines. 624 bp represents the length of the first and second exons and the first intron; 533 bp represents the length of the first and second exons. **e** Relative expression levels of *BoALG10* in the two parent lines and the transgenic individuals at the 10-leaf stage. Asterisks represent a significantly different expression level from that of F0819, as determined using a *t*-test with three repeats (^*^P < .05, ^**^P < .01).

To verify the function of *BoALG10*, we constructed an overexpression vector, *35Spro:BoALG10^S0835^*, containing the coding sequence of *BoALG10* from the inbred line S0835, which produces a smooth leaf margin. The construct was then introduced into the inbred line F0819, which normally produces a feathered leaf margin (Fig. 4a). We successfully generated three individual transgenic plants, OE-1, OE-2, and OE-3, following an *Agrobacterium*-mediated genetic transformation (Fig. 4c).

**Figure 5:**
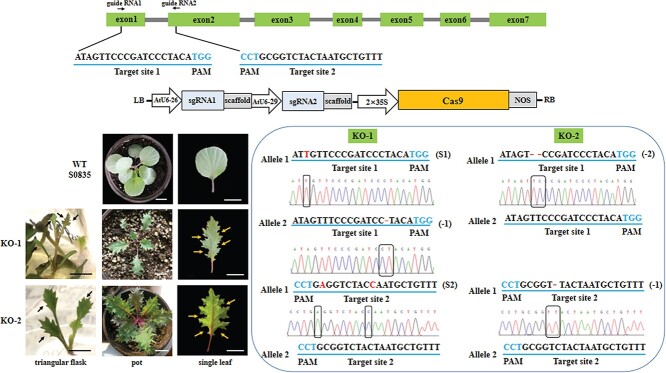
Knockout of *BoALG10* using CRISPR/Cas9 technology. **a** Two single-guide RNA sites were selected in the first and second exons of *BoALG10*. **b** Schematic representation of pHSE401-*BoALG10*, which was used to transform ornamental kale. **c** Phenotype of WT S0835. Scale bars: 2 cm. **d** Phenotypes of the KO-1 and KO-2 knockout lines. Arrows indicate the position of irregular lobes at leaf margin. Scale bars: 2 cm. **e** Editing efficiency of Cas9 at the two target sites. Blue nucleotides represent the PAM, and the red lines or nucleotides indicate different mutations. –, deletion; S, substitution. Black frame indicates mutations. The chromatogram of the WT sequence is shown for reference. The sequences of allele 2 of target site 1 in KO-1, and the allele 2 of target site 1/2 in KO-2 were all the same as the sequences in WT.

We visually inspected the phenotypes of the transgenic individuals and compared them with the wild-type (WT) F0819 (Fig. 4b) and S0835 (Fig. 5c) throughout the growing period. OE-1 produced an ovate blade profile in both the immature and mature leaves, and partially phenocopied the smooth leaf margin of S0835, although it did produce serrations of inconsistent depth (Fig. 4c). The deepest margin lobe occupied two-thirds of the distance from the leaf margin to the main vein, whereas no obvious lobes were produced in either OE-2 or OE-3. The leaf blade of OE-2 was a slightly longer and narrower ovate shape than OE-1, and had an almost S0835-like smooth leaf margin with only a few shallow serrations. OE-3 produced flat and rounded ovate leaves and almost fully phenocopied the smooth leaf margin of S0835, with sporadic shallow serrations. These three strains were thus confirmed to display transgenic phenotypes.

We confirmed the presence of the overexpression cassette in all transgenic plants using PCR (Fig. 4d). The primers were designed to yield a 624-bp PCR product containing the full length of the first and second exons, as well as the first intron, in F0819 and S0835. If the *BoALG10^S0835^* coding region was integrated into F0819, a 533-bp electrophoretic band emerged after amplification, comprising the first and second exons. As expected, two electrophoretic bands emerged at the correct positions for all three of the transgenic strains. The *BoALG10^S0835^* coding sequence was therefore integrated into the F0819 background in these lines.

We detected the relative expression level of *BoALG10* in the transgenic lines (Fig. 4e). The relative expression levels in OE-1 and OE-2 were 2.70- and 6.46-fold higher than in F0819, respectively, while the level was 9.35- and 14.9-fold higher in OE-3 and S0835, respectively, than in F0819; all differences were statistically significant. In summary, we could speculate that *BoALG10* functions to maintain a smooth leaf margin shape.

### Loss of *BoALG10^S0835^* causes irregular dissections in the leaf margin of S0835

We used CRISPR/Cas9-mediated genome editing to generate mutations in *BoALG10* in the S0835 inbred line with smooth leaf margins. Two single target sites were selected in the first and second exons, respectively (Fig. 5a). The single-guide RNA (sgRNA) expression cassette was amplified and then fused into pHSE401, which harbored Cas9 protein (Fig. 5b). The construct was transformed into S0835 via the *Agrobacterium*-mediated method, resulting in two kanamycin-resistant plants, KO-1 and KO-2. Unlike their WT parent (Fig. 5c), which had smooth leaf margins, these two mutants formed irregular lobes at their leaf margins (Fig. 5d). The indentations on the KO-1 leaf margin incorporated serrations of inconsistent depth. The phenotype of KO-2 was similar to KO-1, although it produced a sharper leaf tip. The deepest lobe occupied half of the distance from the leaf margin to the main vein in KO-2.

We amplified the genomic regions on both sides of the target sites (sgRNA1 and sgRNA2) in each transgenic plant to evaluate the mutation(s) that had been introduced into *BoALG10* (Fig. 5e). The two mutants were both heterozygous. The KO-1 mutant contained a single substitution from A to T at the 17th position upstream of the target protospacer-adjacent motif (PAM), a 1-bp deletion in sgRNA1, and two substitutions (C to A and T to C) in sgRNA2 (Fig. 5e); KO-2 contained a 2-bp deletion in sgRNA1 and a 1-bp deletion within sgRNA2. These substitutions and deletions altered the reading frame (Supplementary Data Fig. S1), and the frameshift mutation both in KO-1 and KO-2 presumably led to a non-functional protein. These phenotypes and edited sequences provide further evidence that *BoALG10* functions to maintain the smooth leaf margin morphology.

### Leaf margin cells differed in feathered and smooth leaf margins

**Figure 6:**
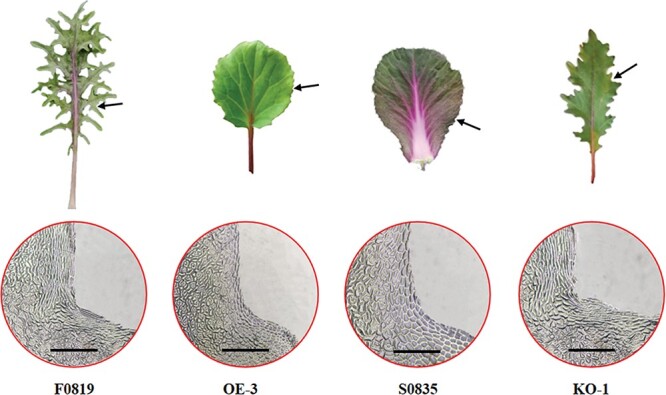
Microscopic observation of epidermal cell morphology in WT and transgenic leaves with different margin shapes. Representative leaf phenotypes of F0819, OE-3, S0835, and KO-1 and corresponding microscopic images of their epidermal cells at the point indicated by the black arrows. Scale bars: 30 μm.

To observe the microscopic differences in leaf margin cell morphology, we captured the epidermal cell shape using nail polish impressions. The epidermal cells showed significant morphological differences in the feathered and smooth leaf margins (Fig. 6). In the feathered leaf margin WT F0819, the epidermal cells were distributed irregularly in the deepest part of the margin indentations. On the contrary, the epidermal cells in the smooth margins were arranged regularly, even in the slight indentations present in S0835 leaves. The cells of the transgenic plant with smooth leaf margins, OE-1, were arranged in a similar manner to those of S0835, while the cell arrangements of the feathered KO-1 leaf margins were similar to those of F0819. The altered cells in the leaf margins might be caused by extrusion among cells in the feathered margins, whereby altered cell arrangements in the irregular lobes may produce different cell morphologies.

## Discussion

### *BoALG10* confers a smooth leaf margin shape

Leaves are the most important organ in plants. Nowadays, much attention is paid to the elucidation of leaf development [6, 20–22, 38, 39]. Several gene families play important roles in leaf margin development, including the *YUCCA* family [13] and the *NAC* family [9, 14, 17]. Leaf lobes are a special margin shape, which are not only a morphological marker in crop breeding but also an important ornamental trait in plants, and have been studied extensively [40–43]. Ni *et al*. [41] constructed overexpression vectors to transform two *AtLMI1* homologs, *BnaA10g26320D* and *BnaA10g26330D*, into WT *A. thaliana*, and *BnaA10g26320D* prompted some lobes to appear at the leaf margin. Thus, *BnaA10g26320D* is the key gene regulating leaf lobe formation in oilseed rape (*Brassica napus*) [41]. In the same year, *LMI1* was also discovered to regulate the leaf lobe trait in upland cotton (*Gossypium hirsutum*) [40]. *BnA10.LMI1* [42] and *BnA10.RCO* [43] both positively regulate the formation of serrated leaf margins in *B. napus*, with *cis*-regulatory variations in these genes leading to the appearance of lobed or serrated leaf margins. In this study, we analyzed the spatiotemporal expression patterns of *BoALG10* in the ornamental kale genotypes F0819 (feathered margin) and S0835 (smooth margin), revealing that it was expressed throughout development in both lines with no differences in tissue specificity. We speculated that the discrepancy of *BoALG10* expression pattern was not sufficient to explain their differences in leaf margin phenotypes. In previous research, we found that two amino acid differences occurred in the conserved domain between F0819 and S0835 [31], and the amino acid variations should be the reason for the phenotype difference.

*ALG10* encodes α-1,2-glucosyltransferase, a key enzyme that catalyzes the formation of adipose-linked oligosaccharides in the N-glycan biosynthesis pathway [28] . The AtALG10 protein was previously reported to be located in the ER, and was found to regulate the dwarf leaf trait in *A. thaliana* [25]. We previously isolated a gene, *BoALG10*, by fine mapping a genomic region with a putative regulatory effect on leaf margin shape in ornamental kale [31]. In the present study, BoALG10 was found to be located in the ER, which was consistent with the subcellular localization of AtALG10 [25].

Using overexpression and CRISPR/Cas9 technology, we verified the function of *BoALG10* in ornamental kale. We therefore conclude that *BoALG10* is responsible for maintaining a smooth leaf margin morphology in ornamental kale, with the mutation of this gene resulting in irregular lobes in the leaf margin.

### Mutated *BoALG10* caused an N-glycosylation defect, resulting in leaf margin dissection

N-glycosylation is a peptide chain modification and a protein co-translational or post-translational modification process [44, 45], beginning in the ER and ending in the Golgi apparatus. N-glycosylation plays a positive role in several developmental aspects, such as plant cell wall formation and the tolerance of abiotic stress [24]. ALG10 transfers the last glucosyl molecule to a liposaccharide precursor to form glucosyl-lipopolysaccharide, which is then transferred to Asn-X-Ser/Thr (where X is any amino acid except Pro) within nascent polypeptides by the oligosaccharide transferase complex STT/OST. Concomitantly, the redundant diphosphate terpene alcohols were released to be recycled in the next oligosaccharide chain assembly. Different STT/OSTs are highly specific for the assembly of different oligosaccharides, and a third glycosyl molecule which was transferred by ALG10 is often necessary for N-glycan biosynthesis [29, 30, 46].

**Figure 7:**
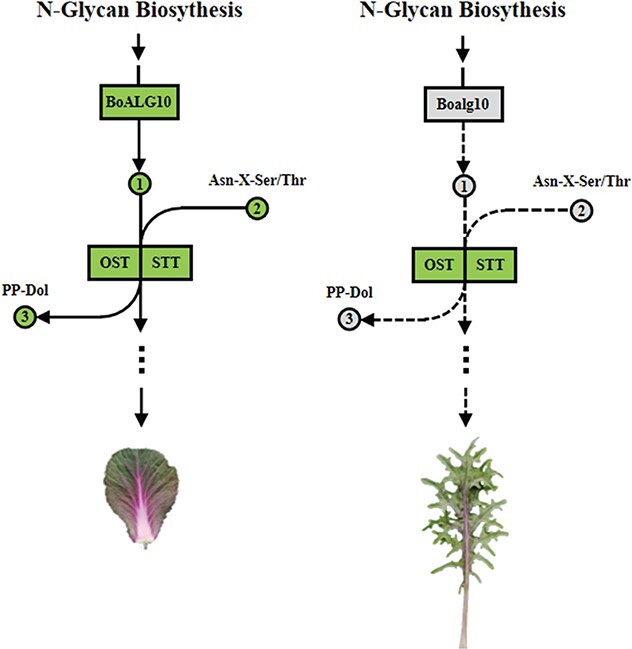
Model of the pathway through which BoALG10 might maintain the smooth leaf margin shape in ornamental kale. Mutation of *BoALG10* (Boalg10) causes a loss of function, blocking the N-glycan biosynthesis pathway, meaning that the smooth leaf margin phenotype cannot be maintained. A gray nodal point indicates that the product cannot be formed, while a green nodal point indicates that the product is formed. Numbers 1–3 in the figure indicate that glucosyl-lipopolysaccharide and nascent polypeptides harbored the Asn-X-Ser/Thr sequence motif and diphosphate terpene alcohols (pp-Dol), respectively.

Previous studies have shown that ALG10 is necessary for N-glycosylation during normal leaf development in *A. thaliana* [25]. The leaf margin was almost smooth and the vein was simple with few secondary vascular veins in *Arabidopsis*. Nevertheless, the leaf vein pattern was very complex with many secondary veins in ornamental kale. Once the gene mutated, leaves became smaller in *Arabidopsis*; in contrast, some lobes appeared in ornamental kale. In *Arabidopsis*, ALG10 is necessary for leaf development, and also in ornamental kale. In the inbred line with a smooth leaf margin, S0835, BoALG10 functioned normally to transfer the last glucosyl molecule to the adipose-linked donor, facilitating N-glycan biosynthesis. The mutation of *BoALG10* in F0819 resulted in its defective function and the disruption of adipose-linked oligosaccharide biosynthesis. The defective N-glycan biosynthetic pathway cannot maintain the smooth leaf margin morphology, and thus irregular lobes appeared in the leaf margin of the mutant ornamental kale (Fig. 7).

Overall, *BoALG10* sustains the smooth leaf margin shape. The dissection of *BoALG10* function could provide new insights into leaf margin morphogenesis. In future studies, we will explore the function of BoALG10 in N-glycan biosynthesis and the mechanism by which the N-glycosylation of downstream proteins affects leaf margin morphology.

## Materials and methods

### Nucleic acid isolation and quality assessment

Genomic DNA was extracted from fresh leaves using the CTAB method [32]. The integrity of the DNA was assessed using electrophoresis on a 1% (w/v) agarose gel on a gel-imaging system (Syngene, Cambridge, UK). The concentration of DNA was adjusted to 50 ng/μL, as determined using a NanoDrop 2000 spectrophotometer (Thermo Fisher Scientific, Waltham, MA, USA).

Total RNA was extracted from fresh tissues collected at different growth stages using the EASYspin Plus Complex Plant RNA Kit (Aidlab, Beijing, China). First-strand cDNA was synthesized with the AMV First-Strand cDNA Synthesis Kit (Sangon Biotech, Shanghai, China).

### Spatiotemporal expression analysis of *BoALG10*

Quantitative real-time PCR (qRT–PCR) was performed as described by Feng *et al*. [31]. Fresh tissues at 10 developmental stages (germinating seeds after 12 and 72 hours, cotyledons, five different true leaves, cauline leaves, and petals) were harvested from the inbred lines F0819 and S0835. All the plants were grown in 2020 in a greenhouse at Shenyang Agricultural University, Shenyang, China, under a photoperiod of 16 hours light/8 hours dark at 25 ± 2°C.

### BoALG10 protein subcellular localization

The full-length coding sequences of *BoALG10* were PCR-amplified using the primers GFP-*BoALG10*F 5′-GAACACGGGGGACTCTTGACATGGGGAGACTAGCGCTTG-3′ and GFP-*BoALG10*R 5′-CCTTTACTAGTCAGATCTACCATCCAAATAAACCTTTGGACACCATC-3′, and then fused with green fluorescent protein (GFP) under the control of the cauliflower mosaic virus *35S* promoter using a NEBuilder HiFi DNA Assembly (New England Biolabs, Ipswich, MA, USA), for which the pCAMBIA1302 vector frame had been digested using the restriction endonuclease NcoI. The recombinant expression vector *35Spro*:*BoALG10*-*GFP* and the ER marker vector *ER-rk* (CD3–959) combined with *mCherry* [33] were introduced into *Agrobacterium tumefaciens* strain GV3101 using the method described by Feng *et al*. [34]. The *35Spro:BoALG10*-*GFP* and *ER-rk* constructs were equally mixed and then infiltrated into 4-week-old *N. benthamiana* leaves, with the pCAMBIA1302 vector used as the blank control. The fluorescent signals were observed under a laser scanning confocal microscope (TCS SP8; Leica Microsystems, Wetzlar, Germany) after a 48-hour incubation in the dark.

### Phylogenetic analysis of BoALG10 protein

Protein sequences homologous to BoALG10 were downloaded from the National Center for Biotechnology Information (NCBI) and used to construct a phylogenetic tree: *Arabidopsis arenosa* (CAE6085553.1), *Arabidopsis lyrata* ssp. *lyrata* (XP_002873050.2), *Arabidopsis suecica* (KAG7553847.1), *A. thaliana* (NP_195861.2), *Brassica cretica* (KAF3568203.1), *B. napus* (XP_013729813.1), *B. napus* (XP_013746118.1), *Brassica oleracea* var. *oleracea* (XP_013623580.1), *Brassica rapa* (XP_009130699.1), *Camelina sativa* (XP_010452078.1), *Capsella rubella* (XP_006287552.1), *Eutrema salsugineum* (XP_006398701.1), *Microthlaspi erraticum* (CAA7021219.1), *Raphanus sativus* (XP_018473028.1), and *Sinapis alba* (KAF8105067.1). ClustalW was used for amino acid sequence alignment, and the phylogenetic tree was constructed using MEGA 7.0 software with the neighbor-joining tree method [35] and 1000 bootstrap replications [36].

### Overexpression of *BoALG10* in the feathered-margined F0819

The plant binary vector pCAMBIA3301 was digested at 37°C for 3 hours in a 50-μL reaction volume comprising 1 μL of the restriction enzymes NcoI and PmlI (New England Biolabs), 10 μL of the pCAMBIA3301 plasmid (50 ng/μL), and 38 μL of RNase-free water. The *BoALG10* coding region from the S0835 inbred line was PCR-amplified from cDNA using the primers F 5′-GGACTCTTGACCATGGGGAGACTAGCGCTTGCAGC-3′ and R 5′-ACCTGTAATTCACACGTGCTACCAAATAAACCTTTGGACACC-3′. The PCR product was inserted between the *35S* promoter and the *Agrobacterium NOS* terminator sequence using the NEBuilder HiFi DNA Assembly (New England Biolabs) to generate the overexpression plasmid *35Spro:BoALG10*. The *A. tumefaciens* GV3101 line containing *35Spro:BoALG10* was introduced into F0819 using the methods described by Feng *et al*. [34].

### Generation of knockout mutants of *BoALG10* by CRISPR/Cas9 technology in the smooth-margined S0835

To further verify the function of *BoALG10*, the CRISPR/Cas9 system was used to generate *boalg10* knockout mutants. The single target sites were selected using the CRISPOR online tool (http://crispor.tefor.net/crispor.py). The *BoALG10*-CRISPR cassette was generated using a PCR amplification from pCBC-DT1T2 using the primers DT1-BsF: ATATATGGTCTCGATTGATAGTTCCCGATCCCTACAGTT, DT1-F0: TGATAGTTCCCGATCCCTACAGTTTTAGAGCTAGAAATAGC, DT2-R0: AACGCGGTCTACTAATGCTGTTCAATCTCTTAGTCGACTCTAC, and DT2-BsR: ATTATTGGTCTCGAAACGCGGTCTACTAATGCTGTTCAA, which were designed as reported previously [37]. The PCR products were digested with BsaI and inserted into pHSE401 using T4 DNA ligase to generate the Cas9 vector pHSE401-*BoALG10*. The PCR fragments were purified and then cloned into the pEASY-T vector (TransGen Biotech, Beijing, China), before being subjected to Sanger sequencing (Sangon Biotech, Shanghai, China). The *A. tumefaciens* GV3101 line containing pHSE401-*BoALG10* was introduced into S0835 using the methods described by Feng *et al*. [34].

### Identification of transgenic and knockout plants

The phenotypes of the transgenic and knockout plants were compared with the WT F0819 and S0835, respectively, in the growing periods. Then, to confirm the integration of the overexpression cassette, specific fragments were PCR-amplified using the primers OEF: 5′-ATGGGGAGACTAGCGCTTGC-3′ and OER: 5′-CACAGCACTGAGGATATATC-3′. The relative transcript levels of BoALG10 were measured in all transgenic plants using the same qRT–PCR method as that described above. To determine the sequences surrounding the target sites, we PCR-amplified using the primers CasF: 5′-TTTGGATTTGACTCCTCTCTG-3′ and CasR: 5′-GATATATCCTCAGTGCTGTG-3′. The PCR fragments were purified and then cloned for sequencing three times as described above.

### Morphological observation of leaf margin cells

A layer of clear nail polish was applied evenly to the adaxial surface of fresh leaf blades from F0819, S0835, OE-3 (a smooth-margined overexpression individual), and KO-1 (a feathered-margined knockout line) plants at the 10-leaf stage. After the nail polish had dried completely, temporary blotting film was used to transfer the nail polish impression of the leaf margin to a microscope slide. The impressions of the leaf margin cells were imaged using an optical microscope (K-700 L; Motic, Hong Kong, China) under 100× magnification.

## Acknowledgements

We are grateful to professor Qijun Chen from China Agricultural University for the CRISPR/Cas9 vectors. This research was supported by the National Natural Science Foundation of China (32171850 and 31770739).

## Author contributions

X.F. and P.Z. designed the experiments. X.F. wrote the manuscript and performed substantial experiments containing nucleic acid extraction, overexpression and CRISPR/Cas9 vector construction, and genetic transformation. X.Y. conducted the subcellular localization of BoALG10 and identified the transgenic plants. M.Z. executed microscopic observation of epidermal cells. X.L. executed spatiotemporal expression analysis of *BoALG10*. P.Z. revised the manuscript. All the authors reviewed and approved this submission.

## Data availability statement

The data that support the results are included in this article and its supplementary materials. Other relevant materials are available from the corresponding author upon reasonable request.

## Conflict of interests

The authors declare that they have no conflicts of interest.

## Supplementary data

Supplementary data is available at *Horticulture Research* online.

## Supplementary Material

supp_data_uhac137

## References

[1] Tsukaya H . Mechanism of leaf-shape determination. *Annu Rev Plant Biol*. 2006;57:477–96. 16669771 10.1146/annurev.arplant.57.032905.105320

[2] Nicotra AB , Leigh A, Boyce CK et al. The evolution and functional significance of leaf shape in the angiosperms. *Funct Plant Biol*. 2011;38:535–52. 32480907 10.1071/FP11057

[3] Dkhar J , Pareek A. What determines a leaf’s shape? *EvoDevo*. 2014;5:47. 25584185 10.1186/2041-9139-5-47 PMC4290414

[4] Vogel S . Leaves in the lowest and highest winds: temperature, force and shape. *New Phytol*. 2009;183:13–26. 19413689 10.1111/j.1469-8137.2009.02854.x

[5] Rowland SD , Zumstein K, Nakayama H et al. Leaf shape is a predictor of fruit quality and cultivar performance in tomato. *New Phytol*. 2020;226:851–65. 31880321 10.1111/nph.16403 PMC7187315

[6] Alvarez JP , Furumizu C, Efroni I et al. Active suppression of a leaf meristem orchestrates determinate leaf growth. *eLife*. 2016;5:e15023. 27710768 10.7554/eLife.15023 PMC5096885

[7] Ori N , Cohen AR, Etzioni A et al. Regulation of *LANCEOLATE* by *miR319* is required for compound-leaf development in tomato. *Nat Genet*. 2007;39:787–91. 17486095 10.1038/ng2036

[8] Shleizerburko S , Burko Y, Benherzel O et al. Dynamic growth program regulated by *LANCEOLATE* enables flexible leaf patterning. *Development*. 2011;138:695–704. 21228002 10.1242/dev.056770

[9] Bilsborough GD , Runions A, Barkoulas M et al. Model for the regulation of *Arabidopsis thaliana* leaf margin development. *Proc Natl Acad Sci USA*. 2011;108:3424–9. 21300866 10.1073/pnas.1015162108 PMC3044365

[10] Li Z , Li B, Shen W et al. TCP transcription factors interact with AS2 in the repression of class-I *KNOX* genes in *Arabidopsis thaliana*. *Plant J*. 2012;71:99–107. 22380849 10.1111/j.1365-313X.2012.04973.x

[11] Bar M , Ori N. Compound leaf development in model plant species. *Curr Opin Plant Biol*. 2015;23:61–9. 25449728 10.1016/j.pbi.2014.10.007

[12] Sarvepalli K , Gupta MD, Challa KR et al. Molecular cartography of leaf development-role of transcription factors. *Curr Opin Plant Biol*. 2019;47:22–31. 30223186 10.1016/j.pbi.2018.08.002

[13] Wang W , Xu B, Wang H et al. *YUCCA* genes are expressed in response to leaf adaxial-abaxial juxtaposition and are required for leaf margin development. *Plant Physiol*. 2011;157:1805–19. 22003085 10.1104/pp.111.186395 PMC3327174

[14] Hibara K , Karim MR, Takada S et al. *Arabidopsis CUC-SHAPED COTYLEDON3* regulates postembryonic shoot meristem and organ boundary formation. *Plant Cell*. 2006;18:2946–57. 17122068 10.1105/tpc.106.045716 PMC1693926

[15] Nikovics K , Blein T, Peaucelle A et al. The balance between the *MIR164A* and *CUC2* genes controls leaf margin serration in *Arabidopsis*. *Plant Cell*. 2006;18:2929–45. 17098808 10.1105/tpc.106.045617 PMC1693934

[16] Hasson A , Plessis A, Blein T et al. Evolution and diverse roles of the cup-shaped cotyledon genes in *Arabidopsis* leaf development. *Plant Cell*. 2011;23:54–68. 21258003 10.1105/tpc.110.081448 PMC3051246

[17] Serra L , Perrot-Rechenmann C. Spatiotemporal control of cell growth by CUC3 shapes leaf margins. *Development*. 2020;147:1–29. 10.1242/dev.183277 32094116

[18] Bresso EG , Chorostecki U, Rodriguez RE et al. Spatial control of gene expression by miR319-regulated TCP transcription factors in leaf development. *Plant Physiol*. 2017;176:1694–708. 29133375 10.1104/pp.17.00823 PMC5813565

[19] Saddic LA , Huvermann B̈, Bezhani S et al. The LEAFY target LMI1 is a meristem identity regulator and acts together with LEAFY to regulate expression of *CAULIFLOWER*. *Development*. 2006;133:1673–82. 16554366 10.1242/dev.02331

[20] Chen J , Yu J, Ge L et al. Control of dissected leaf morphology by a Cys(2)his(2) zinc finger transcription factor in the model legume *Medicago truncatula*. *Proc Natl Acad Sci USA*. 2010;107:10754–9. 20498057 10.1073/pnas.1003954107 PMC2890821

[21] Vlad D , Kierzkowski D, Rast MI et al. Leaf shape evolution through duplication, regulatory diversification, and loss of a homeobox gene. *Science*. 2014;343:780–3. 24531971 10.1126/science.1248384

[22] Kierzkowski D , Runions A, Vuolo F et al. A growth-based framework for leaf shape development and diversity. *Cell*. 2019;177:1405–1418.e17. 31130379 10.1016/j.cell.2019.05.011 PMC6548024

[23] Hajheidari M , Wang Y, Bhatia N et al. Autoregulation of RCO by low-affinity binding modulates cytokinin action and shapes leaf diversity. *Curr Biol*. 2019;29:4183–4192.e6. 31761704 10.1016/j.cub.2019.10.040

[24] Zhang M , Henquet M, Chen Z et al. *LEW3*, encoding a putative α-1,2-mannosyltransferase (ALG11) in N-linked glycoprotein, plays vital roles in cell-wall biosynthesis and the abiotic stress response in *Arabidopsis thaliana*. *Plant J*. 2009;60:983–99. 19732381 10.1111/j.1365-313X.2009.04013.x

[25] Farid A , Pabst M, Schoberer J et al. *Arabidopsis thaliana* alpha1,2-glucosyltransferase (ALG10) is required for efficient N-glycosylation and leaf growth. *Plant J*. 2011;68:314–25. 21707802 10.1111/j.1365-313X.2011.04688.x PMC3204403

[26] Manzano C , Pallero-Baena M, Silva-Navas J et al. A light-sensitive mutation in *Arabidopsis* LEW3 reveals the important role of N-glycosylation in root growth and development. *J Exp Bol*. 2017;68:5103–16. 10.1093/jxb/erx324 29106622

[27] Jiao QS , Niu G-T, Wang F-F et al. N-glycosylation regulates photosynthetic efficiency of *Arabidopsis thaliana*. *Photosynthetica*. 2020;58:72–9.

[28] Burda P , Aebi M. The *ALG10* locus of *Saccharomyces cerevisiae* encodes the α-1,2 glucosyltransferase of the endoplasmic reticulum: the terminal glucose of the lipid-linked oligosaccharide is required for efficient N-linked glycosylation. *Glycobiology*. 1998;8:455–62. 9597543 10.1093/glycob/8.5.455

[29] Karaoglu D , Kelleher DJ, Gilmore R. The highly conserved Stt3 protein is a subunit of the yeast oligosaccharyltransferase and forms a subcomplex with Ost3p and Ost4p. *J Biol Chem*. 1997;272:32513–20. 9405463 10.1074/jbc.272.51.32513

[30] Knauer R , Lehle L. The oligosaccharyltransferase complex from yeast. *Biochim Biophys Acta Gen Subj*. 1999;1426:259–73. 10.1016/s0304-4165(98)00128-7 9878773

[31] Feng X , Li X, Yang X et al. Fine mapping and identification of the leaf shape gene *BoFL* in ornamental kale. *Theor Appl Genet*. 2020;133:1303–12. 31996972 10.1007/s00122-020-03551-x

[32] Murray MG , Thompson WF. Rapid isolation of high molecular weight plant DNA. *Nucleic Acids Res*. 1980;8:4321–6. 7433111 10.1093/nar/8.19.4321 PMC324241

[33] Nelson BK , Cai X, Nebenführ A. A multicolored set of in vivo organelle markers for co-localization studies in *Arabidopsis* and other plants. *Plant J*. 2007;51:1126–36. 17666025 10.1111/j.1365-313X.2007.03212.x

[34] Feng X , Zhang Y, Wang H et al. Dihydroflayonol 4-reductase confers leaf color in ornamental kale. *Theor Appl Genet*. 2021;134:159–69. 33011819 10.1007/s00122-020-03688-9

[35] Saitou NNM , Nei MC. The neighbor-joining method: a new method for reconstructing phylogenetic trees. *Mol Biol Evol*. 1987;4:406–25. 3447015 10.1093/oxfordjournals.molbev.a040454

[36] Kumar S , Stecher G, Tamura K. MEGA7: molecular evolutionary genetics analysis version 7.0 for bigger datasets. *Mol Biol Evol*. 2016;33:1870–4. 27004904 10.1093/molbev/msw054 PMC8210823

[37] Xing H , Dong L, Wang ZP et al. A CRISPR/Cas9 toolkit for multiplex genome editing in plants. *BMC Plant Biol*. 2014;14:327. 25432517 10.1186/s12870-014-0327-y PMC4262988

[38] Kessler S , Neelima S. Shaping up: the genetic control of leaf shape. *Curr Opin Plant Biol*. 2004;7:65–72. 14732443 10.1016/j.pbi.2003.11.002

[39] Israeli A , Capua Y, Shwartz I et al. Multiple auxin-response regulators enable stability and variability in leaf development. *Curr Biol*. 2019;29:1746–1759.e5. 31104930 10.1016/j.cub.2019.04.047

[40] Andres RJ , Coneva V, Frank MH et al. Modifications to a *LATE MERISTEM IDENTITY1* gene are responsible for the major leaf shapes of upland cotton (*Gossypium hirsutum* L.). *Proc Natl Acad Sci USA*. 2017;114:E57–66. 27999177 10.1073/pnas.1613593114 PMC5224360

[41] Ni X , Liu H, Huang J et al. *LMI1*-like genes involved in leaf margin development of *Brassica napus*. *Genetica*. 2017;145:269–74. 28389974 10.1007/s10709-017-9963-0

[42] Hu L , Zhang H, Yang Q et al. Promoter variations in a homeobox gene, *BnA10*.*LMI1*, determine lobed leaves in rapeseed (*Brassica napus* L.). *Theor Appl Genet*. 2018;131:2699–708. 30219987 10.1007/s00122-018-3184-5

[43] Hu L , Zhang H, Sun Y et al. *BnA10*. *RCO*, a homeobox gene, positively regulates leaf lobe formation in *Brassica napus* L. *Theor Appl Genet*. 2020;133:3333–43. 32816057 10.1007/s00122-020-03672-3

[44] Chung CY , Majewska NI, Wang Q et al. SnapShot: N-glycosylation processing pathways across kingdoms. *Cell*. 2017;171:258–258.e1. 28938118 10.1016/j.cell.2017.09.014

[45] Aebi M . N-linked protein glycosylation in the ER. *Biochim Biophys Acta*. 2013;1833:2430–7. 23583305 10.1016/j.bbamcr.2013.04.001

[46] Farid A , Malinovsky FG, Veit C et al. Specialized roles of the conserved subunit OST3/6 of the oligosaccharyltransferase complex in innate immunity and tolerance to abiotic stresses. *Plant Physiol*. 2013;162:24–38. 23493405 10.1104/pp.113.215509 PMC3641206

