# Supplementary material for: BoALG10, an α-1,2 glycosyltransferase, plays an essential role in maintaining leaf margin shape in ornamental kale
Source: Hortic Res. 2022 Jun 15;9:uhac137. doi: 10.1093/hr/uhac137 (PMC9437718; doi:10.1093/hr/uhac137)
Supplement: supp_data_uhac137 [file supp_data_uhac137.zip › Supplemental figures.docx]

**
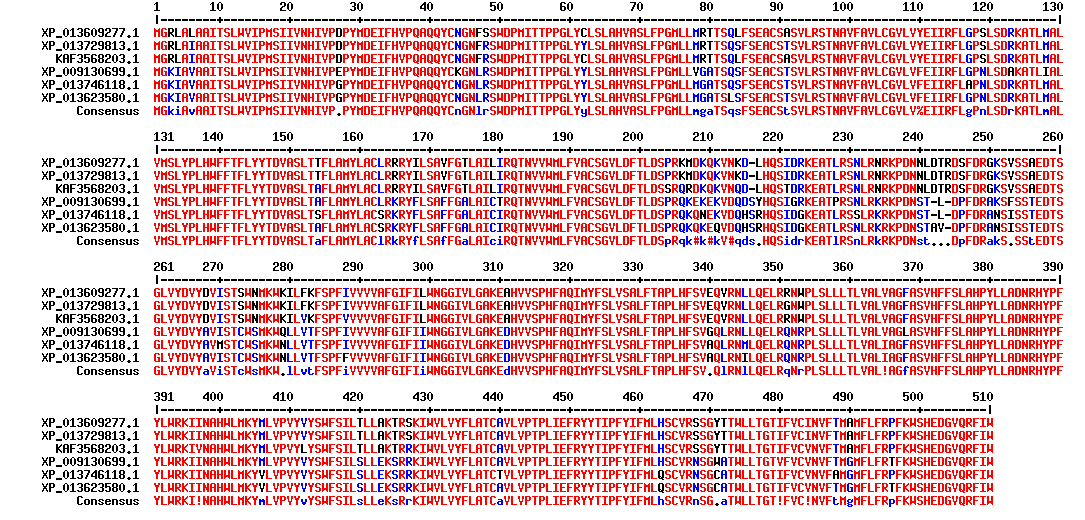
**

**Figure S1** **Protein sequences of the ornamental kale BoALG10 protein and its 5 homologs in *Brassica***

The green frame indicates the conserved domains of these proteins.


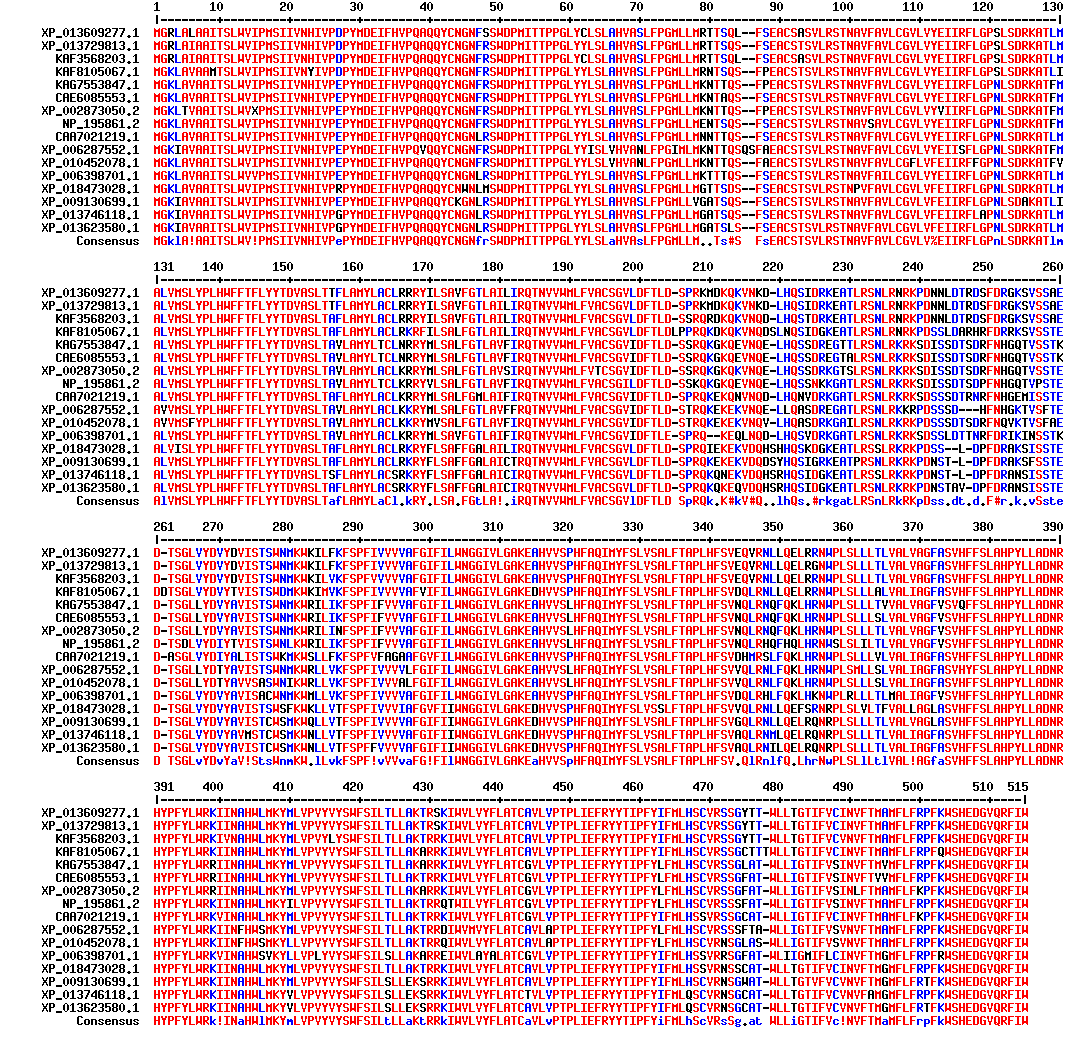


**Figure S2** **Protein sequences of the ornamental kale BoALG10 protein and its 15 homologs in Cruciferae**

The green frame indicates the conserved domains of these proteins.
